# Dysregulation of Intestinal Physiology by Aflatoxicosis in the Gilthead Seabream (*Sparus aurata*)

**DOI:** 10.3389/fphys.2021.741192

**Published:** 2021-12-20

**Authors:** Andre Barany, Milagrosa Oliva, Silvia Filipa Gregório, Gonzalo Martínez-Rodríguez, Juan Miguel Mancera, Juan Fuentes

**Affiliations:** ^1^Department of Biology, Faculty of Marine and Environmental Sciences, Instituto Universitario de Investigación Marina (INMAR), Campus de Excelencia Internacional del Mar (CEI·MAR), University of Cádiz, Cádiz, Spain; ^2^Centre of Marine Sciences (CCMar), Universidade do Algarve, Campus de Gambelas, Faro, Portugal; ^3^Instituto de Ciencias Marinas de Andalucía, Consejo Superior de Investigaciones Científicas (ICMAN-CSIC), Cádiz, Spain

**Keywords:** epithelial barrier, gastrointestinal tract, mycotoxin, tight junctions, Ussing chamber

## Abstract

Aflatoxin B1 (AFB1) is a mycotoxin often present in food. This study aimed to understand the physiological effects of AFB1 on the seabream (*Sparus aurata*) gastrointestinal system. In a first *in vitro* approach, we investigated ion transport using the short-circuit current (I*sc*) technique in Ussing chambers in the anterior intestine (AI). Application of apical/luminal AFB1 concentrations of 8 and 16 μM to healthy tissues was without effect on tissue transepithelial electrical resistance (TER), and apparent tissue permeability (P*app*) was measured using fluorescein FITC (4 kD). However, it resulted in dose-related effects on I*sc*. In a second approach, seabream juveniles fed with different AFB1 concentrations (1 and 2 mg AFB1 kg^−1^ fish feed) for 85 days showed significantly reduced gill Na^+^/K^+^-ATPase (NKA) and H^+^-ATPase (HA) activities in the posterior intestine (PI). Moreover, dietary AFB1 modified I*sc* in the AI and PI, significantly affecting TER in the AI. To understand this effect on TER, we analyzed the expression of nine claudins and three occludins as markers of intestinal architecture and permeability using qPCR. Around 80% of the genes presented significantly different relative mRNA expression between AI and PI and had concomitant sensitivity to dietary AFB1. Based on the results of our *in vitro*, *in vivo*, and molecular approaches, we conclude that the effects of dietary AFB1 in the gastrointestinal system are at the base of the previously reported growth impairment caused by AFB1 in fish.

## Introduction

Fish farming accounted in 2018 for 52% of the total world fish used for food, excluding China, with an estimated production of 82 million metric tons (FAO, 2020). These values have steadily increased over the last decades. However, the present world challenges, such as the decreasing and availability of wild fishery stocks (Tacon and Metian, 2008) and the ongoing climate change, have changed the production paradigm. Therefore, the aquaculture industry is switching to a more sustainable model. The first step to achieve this is the generalized use of plant-based aquafeeds and fish feed additives (Tacon et al., 2011; Daniel, 2018). While this seems the right strategy, it raises new concerns on feed quality, such as pesticide use, market competition, deforestation, and mycotoxins presence.

Mycotoxin contamination and proliferation in aquafeeds represent increasing fish farming concerns (Nácher-Mestre et al., 2015, 2018). Recent numbers estimate contamination by molds and fungi in 25% of the crops (Pandya and Arade, 2016). Some mold species produce mycotoxins (including aflatoxins) as secondary metabolites (Zhang et al., 2014). Liposoluble aflatoxins have low molecular weight *circa* ~0.7 kD (Zhang et al., 2014) and readily cross the skin, and the gastrointestinal epithelium (Ditta et al., 2019). Aflatoxin B1 (AFB1) is the most potent carcinogen and genotoxic among aflatoxins (Flores-flores et al., 2015; Ostry et al., 2017).

Previous studies focused on the dietary effects of AFB1 in farmed fish, including the gilthead seabream (*Sparus aurata*), showed primarily species-specific growth impairment (Santacroce et al., 2012; Gonçalves et al., 2018a,b; Barany et al., 2020). Despite the growth inhibition, there is a lack of apparent pathological signs, and the adverse impact of AFB1 usually goes unnoticed in farmed fish (Anater et al., 2020). Especially interesting is the absence of AFB1 in fish fillets or liver in the gilthead seabream or salmonids after chronic dietary feeding (Nácher-Mestre et al., 2018; Barany et al., 2020), which contrasts with the findings in other species (Huang et al., 2014; Bedoya-Serna et al., 2018) and makes this issue more disturbing. We suggest that the putative absence of AFB1 residue in edible flesh could be due to high AFB1-tolerance implicit in intrinsic-species metabolism (Huang et al., 2011; Deng et al., 2018a,b) or that derived AFB1 bioactive metabolites are responsible for its effects. Such could be the case for AFB1 Exo-8,9-epoxide metabolite (Guengerich et al., 1998), for which commercial detection tests are yet undeveloped.

Anti-nutritional factors present in feeds or the environment (e.g., natural substances or toxicants) impact digestion, the intermediary metabolism, and cause inflammation (Kiron et al., 2020). Many types of anti-nutrient compounds appear in aquafeeds, especially in plant protein-rich feeds. Fermentation, heat treatments, antioxidants inclusion, and clay sequestrants are some methods used for anti-nutrient removal (Estensoro et al., 2016; Chakraborty et al., 2019). The effectiveness of the methods is dependent on the anti-nutritional compound nature. Specifically, several sequestrants or additives have been tested with aflatoxins to counteract their adverse effects (Rodrigues et al., 2019; de Freitas Souza et al., 2020).

Recent studies have pointed to the intestinal epithelium as a potential target of the actions of mycotoxins in humans, specifically in epithelial integrity (Akbari et al., 2017). Intestinal epithelia display two distinctive features: polarity across membranes and tightness among epithelial cells. The asymmetric distribution of proteins that regulate ion movements across the apical and basolateral membranes generates polarity. In contrast, tight junctions (TJs), which are multiprotein junctional complexes, prevent cell-to-cell leakage (Shen et al., 2011) and determine epithelial integrity and selectivity. The TJs permeability dynamic is mediated by claudins and occludins, among other elements (Mukendi et al., 2016). Both integral membrane proteins are the major structural and functional proteins within TJs to form the intercellular seal and permeability control (Claude, 1978; van Itallie and Anderson, 2006, 2013; Zhang and Guo, 2009).

According to their function, claudins are classified as barrier claudins that reduce permeability or pore claudins that promote selective ion permeability (van Itallie and Anderson, 2006; Hou et al., 2013). They can also interact with intracellular signaling molecules, linking extracellular transport, and intracellular signaling events (Collins et al., 2017). More than 63 genes encoding claudins exist in teleost fish (Kolosov et al., 2013; Lu et al., 2013). Of those, 30 claudins show some expression level in the gastrointestinal tract (GIT), but there is no evidence of their function (Tipsmark et al., 2010). The role of occludins is linked to ion selectivity and permeability, based on morphological, physiological, and mRNA expression results on studies performed in frog urinary bladder and in weaning piglet intestine (Claude, 1978; Zhang and Guo, 2009). Overall, information on occludins distribution and physiology is scarce at best.

Our recent work in seabream (*Sparus aurata*) showed that chronic ingestion of AFTB1 impairs growth and metabolism (Barany et al., 2020) as well as the physiological responses to additional stressors, such as crowding densities (Barany et al., 2021). In this line, we hypothesized that the gastrointestinal system is at the base of the previously reported growth impairment caused by AFB1 in fish since it establishes the first contact with food and its components. Therefore, this study aimed to characterize the direct effects of AFB1 on intestinal physiology by 1) comparing electrophysiological properties *in vivo* vs. *in vitro* expositions (i.e., short vs. long-term); 2) the morphological modifications caused *in vivo* after long-term exposure; and 3) the mRNA expression alterations *in situ* on putative proteins that keep cell-to-cell adhesion along with the gastrointestinal architecture.

## Materials and Methods

### Animals

Seabream juveniles (*Sparus aurata*, 100–200 g body mass) were obtained from the local stock of Ramalhete Marine Station (CCMar, Faro, Portugal) for the *in vitro* experiments or purchased from CUPIBAR SL, Cádiz, Spain for the *in vivo* experiments.

### Series 1: AFB1 in Control Tissue *in vitro*

This series of experiments intended to clarify if the effect of AFB1 on the intestine was immediate or required of chronic exposure. Tissues from control fish (not exposed previously to AFB1) were collected and mounted in Ussing chambers (see below). The concentrations of AFB1 used (8 and 16 μM) were based on estimations of minimum and maximum presence of this compound *in vivo* that would leach from the feeding experiments. For these estimations, fish size, food ration, luminal fluid volume, and the content of AFB1 in the feeds were taken into account (Gilannejad et al., 2019; Barany et al., 2020).

### Short-Circuit Current (I*sc*)

After anesthesia (1 mL 2-phenoxyethanol/L seawater) and decapitation, the anterior intestine was collected fresh, isolated, and mounted in Ussing chambers, as previously described (Gregorio et al., 2012). Tissues were open longitudinally, flattened, placed on a tissue holder of 0.25 cm^2^, and positioned between two half-chambers containing 2 mL of serosal physiological saline (e.g., symmetrical conditions) formulated for seabream (Fuentes et al., 2006), in mM: 160 NaCl, 1 MgSO_4_, 2 NaH_2_PO_4_, 1.5 CaCl_2_, 5 NaHCO_3_, 3 KCl, 5.5 Glucose, Hepes 5 mM; 300 mOsm/kg H_2_O and pH to 7.80). *In vitro*, the saline received a gas mixture of 99.7% O_2_ + 0.3% CO_2_, and the temperature was kept constant at 18°C according to the temperature maintained in the tanks with the fish when alive.

*In vitro* preparations were left undisturbed until reaching a steady state. After 1 h, enough AFB1 was sequentially added at 1-h intervals to give a final concentration of 8 and 16 μM, respectively. AFB1 was added apically to simulate leaching from food. Transepithelial electric resistance (TER, Ω cm^2^) calculations used the current deflections induced by a ± 1 mV pulse of 3 s every minute. Voltage clamping and current injections were performed using epithelial amplifiers VCC600 (Physiologic Instruments, San Diego, United States). All data were recorded onto a computer using a Lab-Trax-4 acquisition system (World Precision Instruments, Sarasota, FL, United States) using LabScribe3 (iWorx Systems Inc., Dover, NH, United States).

#### Intestinal Permeability

After tissue stabilization *in vitro*, saline solutions were replaced with new well-gassed solutions. Enough Fluorescein isothiocyanate–dextran (FITC, average mol wt ~4,000, Sigma, Madrid) prepared as concentrated stocks of 100 mg/mL were added to final concentrations of 0.5 mg/mL to the apical chamber. A sample (0.2 mL) was collected from both the apical and the basolateral compartments at time zero. Note that previously chambers were let for 15 min mixing with the oxygen bubbling in order to homogenize the serosal saline and the added FITC. New samples from both the donor and receiver compartments were collected into fresh vials after a 1-h control period (starting from time zero) and the subsequent 1-h periods in the presence of apical AFB1 (8 and 16 μM). The samples were stored at −20°C until analysis.

Fluoresce measurements were performed using a Multi-Mode Microplate Reader BioTek Synergy™ 4 (BioTek® Instruments, Winooski, VT, United States) set for excitation wavelength at 492 nm and emission wavelength at 520 nm for FITC. Standard concentrations were between 0.2 and 2000 ng/mL to calculate concentrations in both the apical and basolateral chambers. The apparent permeability (P*app*) was estimated using the equation (Arnold et al., 2019): Papp = (dC/dT) x (V/A*C_0_) where P*app* is the permeability in centimeters per second; dC/dT is the rate of concentration change (in ng/s) of FITC in the receiving chamber (basolateral) calculated from the slope of the concentration-time curve between 15 and 75 min; V is the volume of the receiver chamber in mL; A is the surface area of the tissue in square centimeter; and C_0_ is the starting concentration in the donor compartment (apical).

All animal experimentation was carried out in compliance with the European (Directive 2010/63/EU) and Portuguese legislation for the use of laboratory animals. All animal protocols were performed under Group-C licenses from the Direção-Geral de Alimentação e Veterinária, Ministério da Agricultura, do Mar, Ambiente e Ordenamento do Território, Portugal.

### Series 2: *in vivo* AFB1 Feeding

The influence of different dietary doses of AFB1 (1 and 2 mg AFB1 kg^−1^ fish feed) during 85 days on gilthead seabream (*Sparus aurata*) juveniles was previously assessed (Barany et al., 2020). Briefly, fish (176.79 ± 2.52 g body mass and 19.16 ± 0.09 cm furcal length) were acclimated to our facility at Servicio Central de Investigación en Cultivos Marinos (SCI-CM, CASEM, University of Cadiz, Spain; Operational Code REGA ES11028000312). Fish were homogenously distributed, in triplicates, in nine 500 L-fiber glass tanks continuously aerated in an open circuit flow-through seawater system. In each tank, the number of individuals was adjusted to achieve the same experimental density (4 g L^−1^; Barany et al., 2020, 2021) and number (n = 30) in the three experimental groups. Fish were fed for 85 days with: (i) fish feed without AFB1 (CT); (ii) 1 mg AFB1 kg^−1^ fish feed (D_1_); and (iii) 2 mg AFB1 kg^−1^ fish feed (D_2_). Parameters of the water, such as oxygen concentration, pH, and salinity, were analyzed daily (data not shown), and a 33% tank water was replenished daily to ensure good water quality. Fish were fed three times a day (distributed throughout the daylight) at 1.5% of their body mass using Eheim 3,581 Feed-Air digital automatic feeders (EHEIM GmbH & Co KG, Germany). All the experimental procedures followed the guidelines of the University of Cádiz (Spain) and the European Union (Directive 2010/63/EU) for the use of animals in research which were also previously approved (Junta de Andalucía reference number 28-04-15-241) by the Ethics and Animal Welfare Committee from the Spanish Government (RD53/2013).

#### Experimental Fish Feed

A commercial fish feed (Skretting, Burgos, Spain) was used as a basis to prepare the experimental diets to supply all essential nutritional requirements for the gilthead seabream (57% crude protein, 18% crude fat, 10% ash, 1.6% phosphorus, and 19.5 MJ kg^−1^ digestible energy). The inclusion of AFB1 (Sigma A6636) at levels of 0 (CT), 1 (D_1_), and 2 (D_2_) mg kg^−1^ fish feed was performed after grounding and re-pelleting. Prof. Francisco Javier Moyano’s Research group, from the University of Almería, made the experimental aquafeeds by extrusion at the Experimental Feed Service facilities.

#### Sampling Protocol

Fish were euthanized under anesthesia of 1 mL 2-phenoxyethanol L^−1^ seawater and sampled. Blood was collected from the caudal peduncle into 1 mL syringes rinsed with a solution containing 8,000 Units mL^−1^ 0.9% NaCl (heparin ammonium salt, Sigma H6279) then fish were beheaded. Plasma was obtained by blood centrifugation (10,000 *g*, 3 min at 4°C), frozen in liquid nitrogen, and stored at −80°C until analysis. The second gill arch from both dorsal sides was excised, adherent blood was removed by blotting with absorbent paper, and biopsies of each branchial arch, consisting of a few filaments, were cut using a surgical blade.

Biopsies of intestinal mucosa were collected with fine-point scissors. Two sections of the intestine were distinguished: (1) anterior intestine, corresponding to 2–3 cm caudal to the stomach, and (2) the posterior intestine, corresponding to a section of 3–4 cm in length of distal intestine, delimited in the end by the rectal sphincter. Gill and intestinal biopsies were placed in ice-cold sucrose-EDTA-imidazole (SEI) buffer (150 mM sucrose, 10 mM EDTA, 50 mM imidazole, pH 7.3) and frozen in liquid nitrogen to estimate ATPase activity. Representative biopsies of the anterior intestine and posterior intestine were placed in RNAlater (Ambion®, Applied BioSystems). These samples were kept for 24 h at 4°C and then stored at −20°C until RNA isolation. Control and experimental groups were randomly sampled from tanks at random time points during each experiment to minimize tank effects.

#### Osmolality and Sodium

Plasma osmolality was measured in 20 μl samples with a Vapro 5,520 Osmometer (Wescor, United States). Sodium was measured by a flame photometer (BWB-XP Performance Plus, BWB Technologies, United Kingdom).

#### ATPases Activities

Gill and intestinal NKA and HA activities were analyzed using an NADH-linked kinetic assay in a 96-well microplate run at 25°C for 10 min, as described in McCormick (1993). Frozen tissues were homogenized on ice-cold SEID (0.1% sodium deoxycholate in SEI buffer, pH = 7.3) and centrifuged at 3,200 *g* for 5 min at 4°C. The supernatant was assayed for ATPase activity in the presence and absence of the NKA-specific inhibitor ouabain (0.5 mM) and HA-specific inhibitor bafilomycin A1 (100 nM), as previously described (Ruiz-Jarabo et al., 2017). NADH oxidation was determined spectrophotometrically at 340 nm. The difference in kinetics between the inhibited and uninhibited assay mixtures was used to calculate NKA and HA-specific (ouabain/bafilomycin A1-sensitive). The activity was expressed as μmol ADP/mg protein/h. Total protein was measured using the bicinchoninic acid protein assay (BCA) with a bovine serum albumin (BSA) standard (Thermo Scientific, Rockford, IL, United States). The assay was run on an automated microplate reader (PowerWave 340, BioTek Instrument Inc., Winooski, United States) controlled by KCjunior™ software.

#### Short-Circuit Current (I*sc*)

On sampling days, the anterior and posterior intestine were collected fresh, isolated, and mounted in Ussing chambers, as described for Series 1. Tissues were left undisturbed until I*sc* readings achieved a steady state, usually ~30 min after mounting. The transepithelial potential difference (TEP, in mV) was referenced to the apical side. Short-circuit current (I*sc*, μAmp/cm^2^) was monitored by clamping epithelia to 0 mV and expressed as negative for the absorption of anions. Transepithelial electric resistance (TER, Ω cm^2^) was calculated using the current deflections induced by a + 1 mV pulse of 3 s every minute using Ohm’s law. Voltage clamping and current injections were performed using epithelial amplifiers DVC1000 (World Precision Instruments, Sarasota, FL, United States). All data were recorded onto a computer using a Lab-Trax-4 acquisition system (World Precision Instruments, Sarasota, FL, United States) using LabScribe3 (iWorx Systems Inc., Dover, NH, United States).

#### RNA Isolation and Quantitative Real-Time PCR

Biopsies from the anterior and posterior intestines were individually processed. Tissues were homogenized using an Ultra-Turrax® T25 with an S25N-8G dispersing tool (IKA®-Werke), and total RNA was extracted using the NucleoSpin® RNA kits (Macherey Nagel) following the manufacturer instructions. Genomic DNA (gDNA) was removed *via* on-column DNase digestion at 37°C for 30 min using rDNase (RNase-free) included with the kits. RNA concentration was measured with a Qubit 2.0 fluorimeter and Qubit RNA BR assay kit (Life Technologies). RNA quality was assessed using a Bioanalyzer 2,100 with the RNA 6000 Nano kit (Agilent Technologies). Only those samples with an RNA integrity number greater than 8.0 were used in real-time quantitative PCR (qPCR). Total RNA (500 ng) from each sample was reverse-transcribed in a 20 μl reaction using the qScript™ cDNA synthesis kit (Quanta BioSciences) in a Mastercycler® proS (Eppendorf), as previously described by Mata-Sotres et al. (2016). A pool of cDNAs from all the samples for each tissue was used to construct calibration plots, using serial 1:10 dilutions from 10 ng to 100 fg, to assess qPCR linearity and efficiency as for use for inter-assay calibrations. RNase-free water (NTC) and RNA (NRT) were included in the analysis as control reactions to ensure the absence of primer-dimers and genomic DNA contaminations, respectively. For all primers pairs, amplification linearities (R^2^) and efficiencies ranged from 0.980–0.999 and 0.925–1.087, respectively. Reactions for qPCR were performed in triplicate with 10 ng of cDNA (estimated from the input of total RNA), forward and reverse primers for the named samples (optimum 200 or 400 nM each), and PerfeCTa™ SYBR® Green FastMix™ (Quanta BioSciences). Reactions were performed in a volume of 10 μl using Hard-Shell® Low-Profile Thin-Wall 96-Well Skirted PCR plates (Bio-Rad) covered with Microseal® B Adhesive Seals (Bio-Rad). Relative gene expressions were quantified in a CFX Connect™ Real-Time PCR Detection System under the control and analysis of CFX Manager™ software (Bio-Rad) using the ΔΔC_T_ method (Livak and Schmittgen, 2001), corrected for efficiencies (Pfaffl, 2001). The amplification included an initial denaturation and polymerase activation step at 95°C for 10 min, followed by 40 cycles of denaturation for 15 s at 95°C, annealing, and extension at 60°C for 30 s. After amplification, individual melting curves from 60°C to 95°C (0.5°C every 5 s) were generated to confirm single amplicons and the absence of primer-dimer artifacts. Results were normalized to the geometric average expression of *eef1a* and *actb* (Vandesompele et al., 2002). The genes were selected due to their lower than 0.5 target stability M value and lower than 0.25 CVs (as indicated by BioRad CFX Manager Target Stability Value). Table 1 shows the primer sequences used for qPCR and the resulting amplicon sizes. Sequence confirmation of amplified fragments was performed by Sanger sequencing (CCMar Sequencing Facility). Claudin and occludin sequences were retrieved and identified from an in-house seabream intestinal transcriptome assembly (Fuentes Lab). Please refer to Supplementary Material for further details.

**Table 1:** Specific primers used for real-time qPCR expression analyses, primers final concentration (nM), GenBank accession numbers, and sizes of the amplified products in base pairs (bp).

Genes	Nucleotide sequence (5′ → 3′)	nM	Accession. no.	bp
*cldnb* (*)	F: CCACACCATCATCCGAGAC R: CTCATCTTTAGGAGGGCAGTTG	200	MW731470.1 XM_030437103.1	151
*cldnk* (*)	F: CTCTGGCTCTGGGTGTCCTC R: CTGATGATGGAGTGGGCAGT	200	MW731472.1 XM_030408301.1	154
*cldn3*	F: TGAGGGTGAACTGAGGAACA R: TGGAAGACAAAGAGCCTACG	200	KF861991.1 XM_030440595.1	115
*cldn5*	F: CTGCTGTGCTGCTCCTGTC R: GTTCTGCGTGGCTCTCTTG	400	MW731471.1 XM_030418403.1	81
*cldn7b*	F: TACGCTCACGACATCATCCA R: CCAACTACAGCCAGGAAAGC	200	MW731473.1 XM_030439139.1	116
*cldn12*	F: CCAGTTTACATTGCCTTTGTTC R: ATCTGCATTCTCATTCCTGAA	200	KF861992.1 XM_030393069.1	180
*cldn15*	F: AAACCCACTTTGTGATTGCA R: TGTTTGACCTTCCCCTTACAA	200	KF861993.1 XM_030431412.1	154
*cldn24*	F: GGGATGCTGGGAATGCTG R: AAAGTCCTCTTGGTGCGAAA	200	MW731474.1 XM_030432503.1	83
*cldn34*	F: CTCCGCAGTCTCAACACGT R: CACAGGTAGATCCAGGTGCA	200	MW731475.1 XM_030409841.1	158
*ocln1* (*)	F: AGAAACAGGCAATGAACTCG R: GGTCGGCGTCAAACTCTCT	200	KF861990.1 XM_030434673.1	104
*ocln2* (*)	F: TGTTGTTGTTTCGGAGAGAGC R: CGACGACTGTTCTTGTCAGC	200	MW731476.1 XM_030433803.1	138
*ocln3* (*)	F: TCGCGTTGTTGATGCTAATA R: TGGTTGACGAACACTCCTGA	200	JQ692876.1 XM_030403521.1	148
*actb*	F: TCTTCCAGCCATCCTTCCTCG R: TGTTGGCATACAGGTCCTTACGG	200	X89920.1 XM_030406939.1	108
*eef1a*	F: AGAGGCTGTCCCTGGTGA R: TGATGACCTGAGCGTTGAAG	200	AF184170.1 XM_030411990.1	137

(*) See Supplementary Material for further details (Supplementary Figures S1, S2).

#### Histopathological Analysis

Intestinal samples collected from the CT and D_2_ groups (n = 9) were used for the analysis. The histological tissue samples were fixed in phosphate-buffered saline at pH 7.2 containing formalin (10%). Then the preparations were washed in running tap water, dehydrated in alcohol, cleared in xylene, and embedded in paraffin wax. Sections (6 μm) were cut and mounted on gelatinized slides using a rotary microtome. Sections were rehydrated in distilled water and stained with hematoxylin/eosin (H&E). Prepared slides were then examined and photographed (Jenoptik ProgRes CT5) under a light microscope (Nikon eclipse Ci-L).

### Chemicals

All chemicals were purchased from Sigma-Aldrich (Madrid, Spain). Ouabain (03125) and bafilomycin A1 (B1793) were prepared as concentrated stocks in imidazole buffer (50 mM). Concentrated stocks of aflatoxin B1, AFB1 (A6636), and fluorescein isothiocyanate-dextran, FITC (46945) were prepared in dimethyl sulfoxide (DMSO). When added to the Ussing chambers, the final volume of DMSO never exceeded 0.5% of the total volume of mucosal saline. DMSO did not show an effect on tested control tissues (data not shown).

### Statistical Analysis

All data are presented as the mean ± standard error of the mean (SEM). Statistical comparison for all given results was performed using ANOVA, after assessing normality and equal variance using Shapiro–Wilk and D’Agostino-Pearson tests, respectively. For all data sets, outliers were identified by the ROUT method at Q = 1%. All ANOVA analyses were followed either by Tukey’s or Bonferroni *post hoc* tests to identify specific significant differences. All statistical analyses were performed with GraphPad Prism 6.0 (GraphPad Software, La Jolla, CA, United States), and significance for all tests was set at *p* < 0.05.

## Results

Dietary AFB1 did not significantly change plasma osmolality or Na^+^ levels (Table 2).

**Table 2:** Plasma osmolality and sodium in *S. aurata* juveniles fed with different experimental diets (CT, D_1,_ and D_2_) for 85 days.

Parameters	CT	AFB1 (D_1_)	AFB1 (D_2_)
Osmolality (mOsm kg^−1^ H_2_O)	370.8 ± 2.7	349.1 ± 9.9	361.3 ± 3.5
Sodium, Na^+^ (mM)	169.1 ± 2.8	175.2 ± 3.2	176.4 ± 3.1

*Data are presented as mean ± SEM (n = 9). Letters indicate significant differences (p < 0.05, one-way ANOVA followed by Tukey’s post hoc analysis)*.

### Gill and Intestinal ATPase

**Figure 1:**
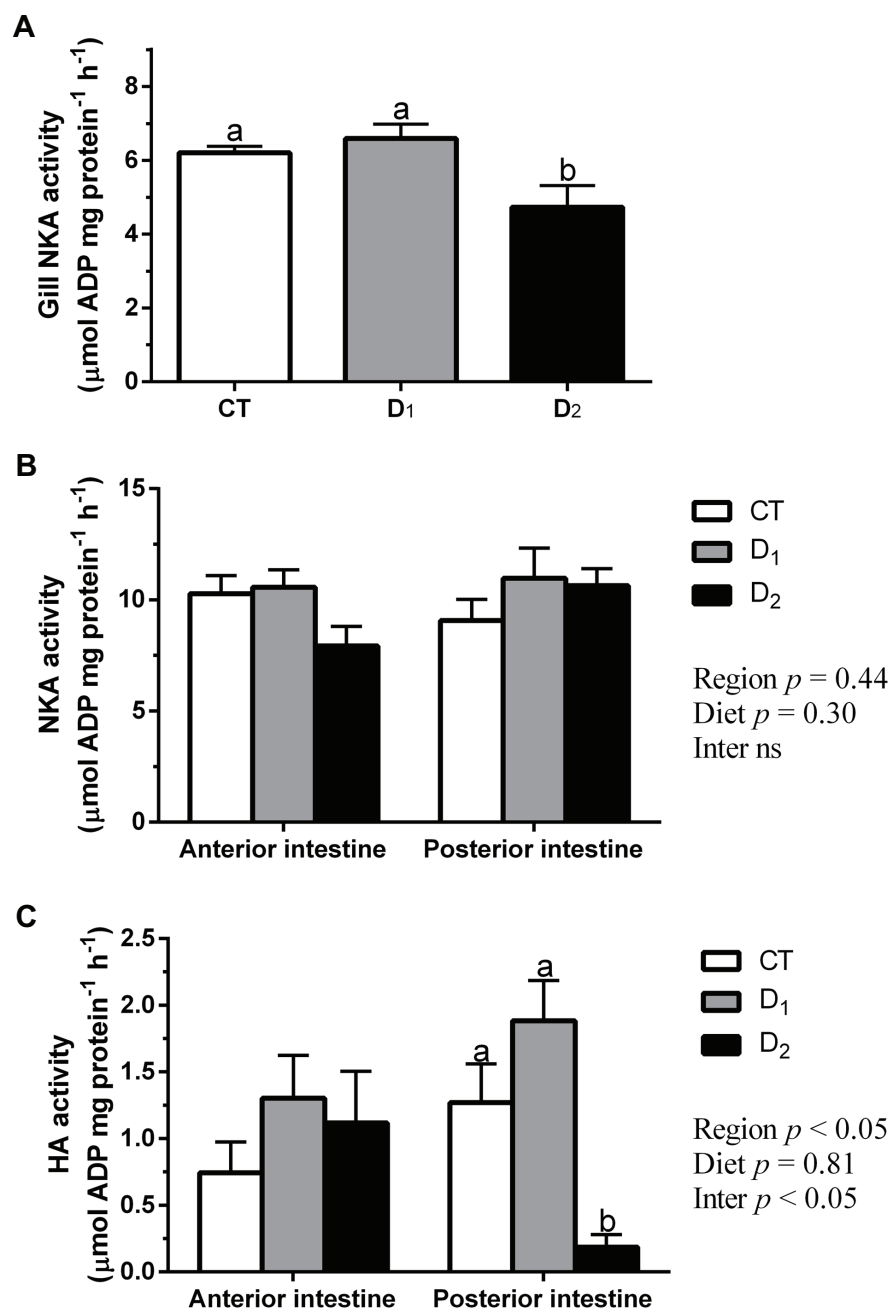
Gill NKA activity **(A)**, intestinal NKA activity **(B)**, and intestinal HA activity **(C)** in *S. aurata* juveniles fed with different experimental diets (CT, control; D_1_, 1 mg-; and D_2_, 2 mg AFB1 kg^−1^ fish feed) for 85 days. Data are presented as mean ± SEM (n = 6–10). Different letters indicate significant differences among the same intestinal regions. Abbreviation “Inter ns”: interaction not significant (*p* < 0.05, one or two-way ANOVA followed by Tukey’s *post hoc* analysis).

Gill NKA activity decreased significantly in the D_2_ group compared to CT and D_1_ groups (Figure 1A). In contrast, no significant differences were observed in NKA activity, neither in the anterior nor posterior intestine, although absolute activity values were lower in group D_2_ in the anterior intestine (Figure 1B). Additionally, HA activity showed no significant changes in the anterior intestine. However, this activity was significantly lower in the D_2_ group in the posterior intestine than in the control group or group D_1_ (Figure 1C).

### Dietary Effect of AFB1 on Intestinal Bioelectrical Properties

#### Series 1: AFB1 in Control Tissue *in vitro*

**Figure 2:**
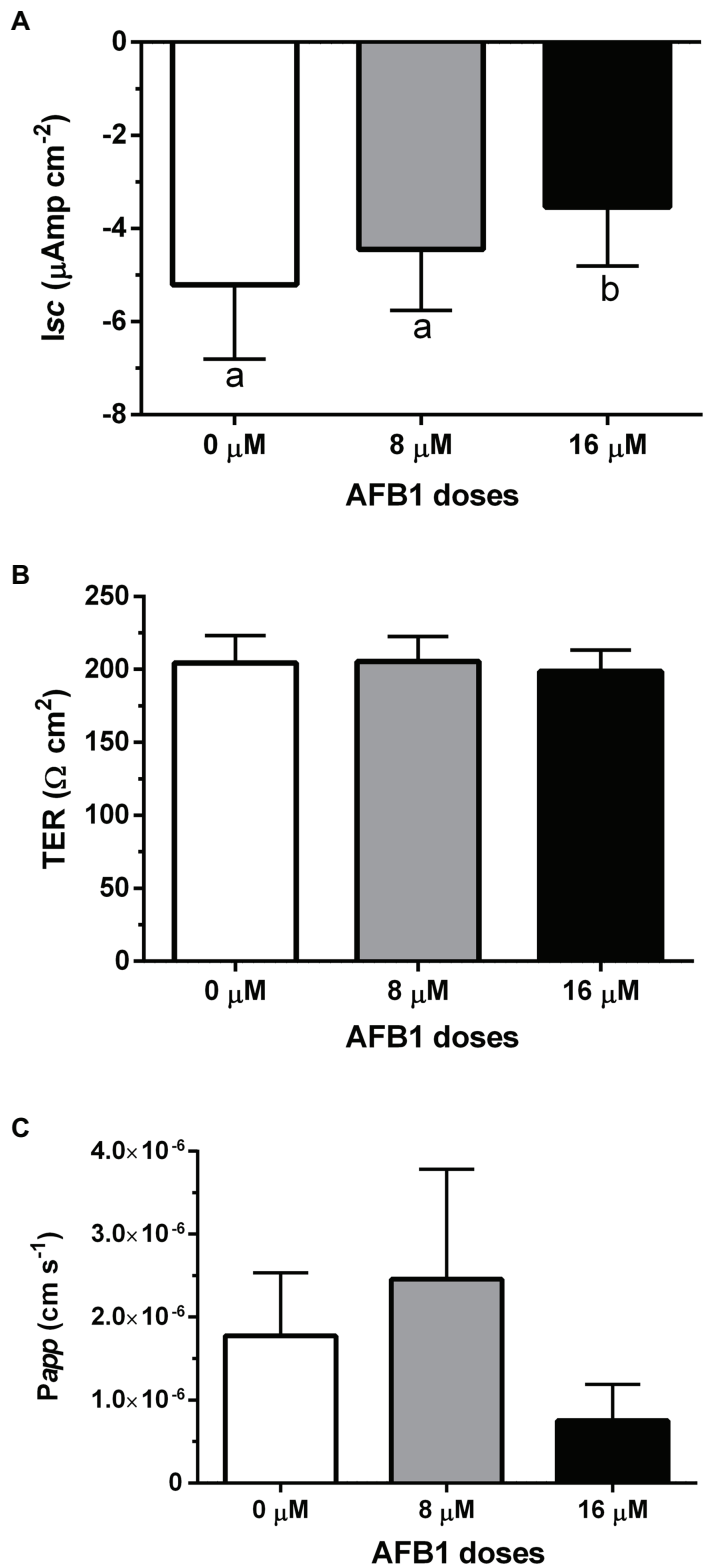
I*sc* **(A)**, TER **(B)**, and P*app* **(C)** in response to increasing concentrations of luminal AFB1 (8 and 16 μM) in the anterior intestine of control *S. aurata* individuals mounted in Ussing chambers. Data are presented as mean ± SEM (n = 6; *p* < 0.05, matched-measures one-way ANOVA followed by Tukey’s *post hoc* analysis).

Under voltage clamp to 0 mV and symmetric conditions, the anterior intestine of the control seabream group mounted in Ussing chambers had an absorptive short-circuit current (I*sc*, μAmp cm^−2^) of −5.21 ± 1.59. However, after AFB1 (8 and 16 μM) was sequentially added at 1-h intervals, I*sc* became −4.45 ± 1.31 and −3.54 ± 1.26, respectively (Figure 2A). In contrast, neither the transepithelial electrical resistance (TER, Ω cm^2^) nor the apparent permeability (P*app*) in the anterior intestine was significantly modified in any group in response to luminal addition of AFB1 (Figures 2B,C).

#### Series 2: *in vivo* AFB1 Feeding

**Figure 3:**
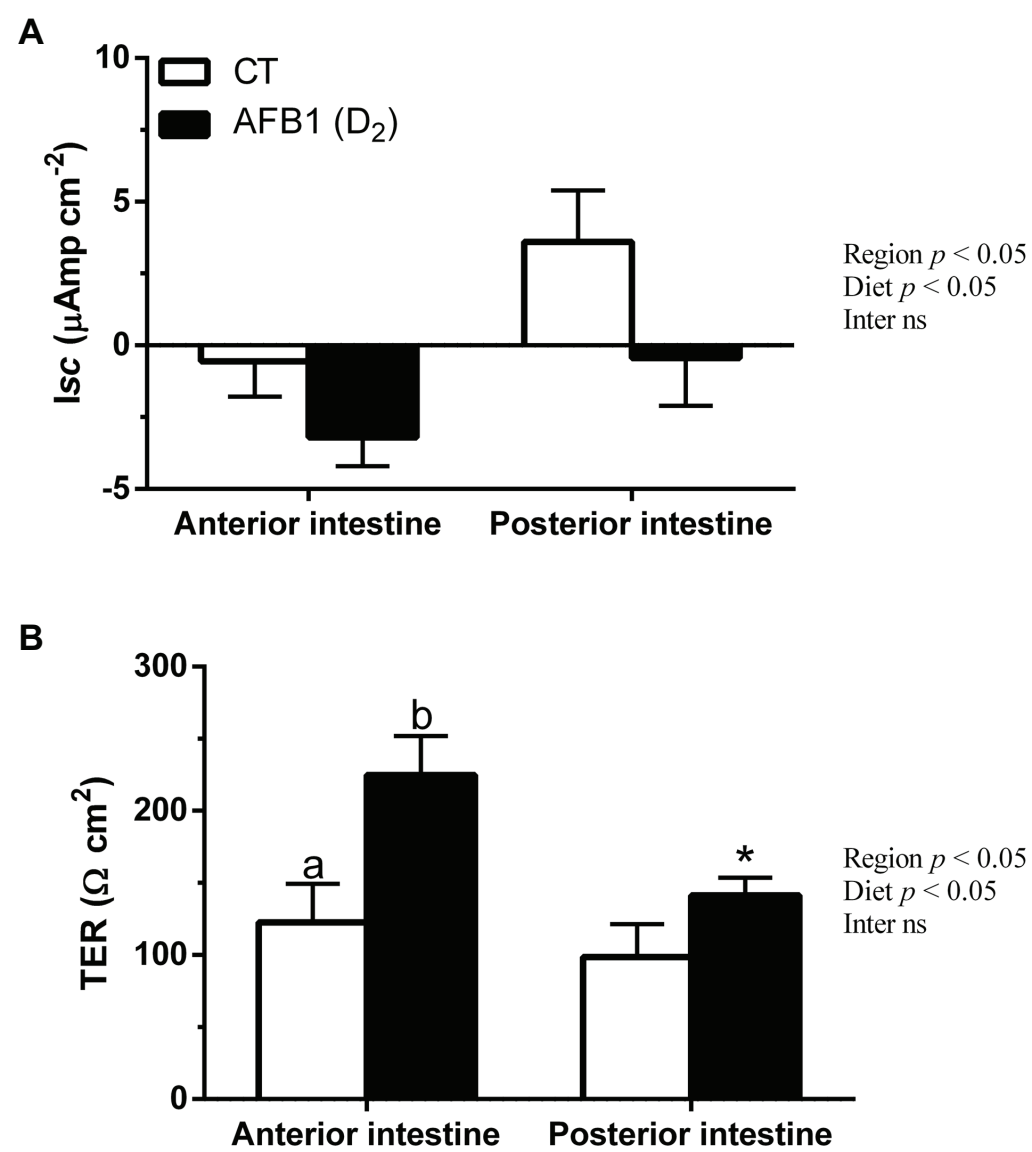
I*sc* **(A)** and TER **(B)** in anterior (AI) and posterior intestine (PI) of *S. aurata* juveniles fed with different experimental diets (CT, control; and D_2_, 2-mg AFB1 kg^−1^ fish feed) for 85 days. Data are presented as mean ± SEM (n = 8–11). For a given intestinal region, different letters indicate significant differences between experimental diets. Asterisks (*) indicate the significance level between different intestinal regions within the same experimental diet. Abbreviation “Inter ns”: interaction not significant (*p* < 0.05, two-way ANOVA followed by Tukey’s *post hoc* analysis).

The anterior intestine of the control seabream group mounted in Ussing chambers had, under voltage clamp to 0 mV and symmetric conditions, an absorptive short-circuit current (I*sc*, μAmp cm^−2^) of −0.56 ± 1.23, while in the posterior intestine, this I*sc* was secretory (+3.60 ± 1.78). In fish from group AFB1 (D_2_), I*sc* was −3.20 ± 1.00 in the anterior intestine and −0.45 ± 1.66 μAmp cm^−2^ in the posterior intestine (Figure 3A).

Transepithelial electrical resistance (TER, Ω cm^2^) was ~123 Ω cm^2^ in the control seabream group’s anterior intestine and was ~99 Ω cm^2^ in the posterior intestine. However, the presence of AFB1 in the feed (group D_2_) significantly increased TER up to ~225 Ω cm^2^ in the anterior intestine while remained statistically unaffected in the posterior intestine (up to ~141 Ω cm^2^). Moreover, AFB1 in the feed highlighted significant differences between the anterior and posterior intestines (Figure 3B).

##### Gene Expression for Claudins and Occludins

**Figure 4:**
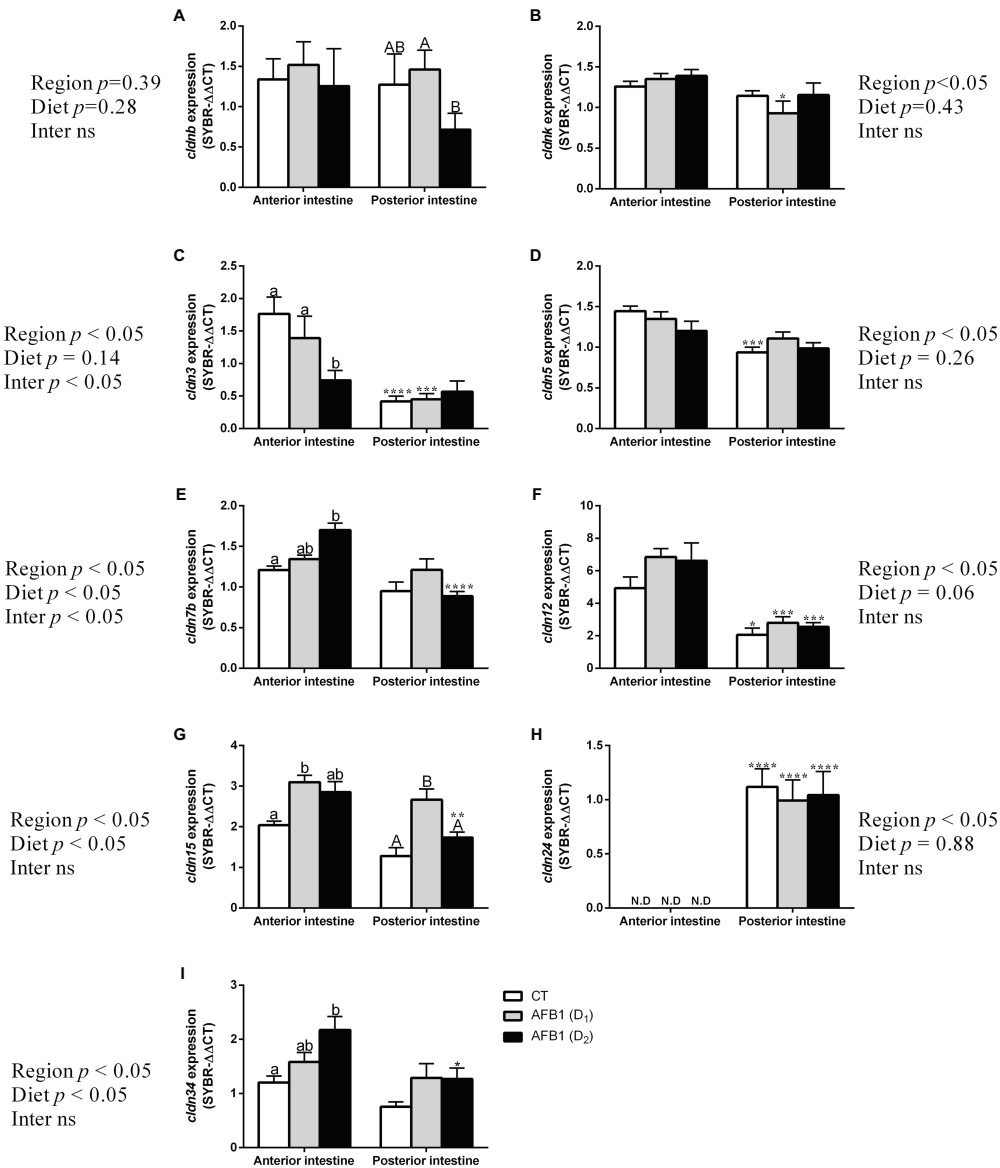
Gene expression of *cldnb* **(A)**, *cldnk* **(B)**, *cldn3* **(C)**, *cldn5* **(D)**, *cldn7b* **(E)**, *cldn12* **(F)**, *cldn15* **(G)**, *cldn24* **(H)**, and *cldn34* **(I)** in anterior (AI) and posterior intestine (PI) of *S. aurata* juveniles fed with different experimental diets (CT, control; D_1_, 1 mg- and D_2_, 2 mg-AFB1 kg^−1^ fish feed) for 85 days. Data are presented as mean ± SEM (n = 7–10). Different letters indicate significant differences among groups within the same intestinal region (lowercase letters: AI; capital letters: PI). Asterisks (*) indicate the significance level between different intestinal regions within the same experimental diet; Abbreviations, “ND”: not detected; “Inter ns”: interaction not significant (*p* < 0.05, two-way ANOVA followed by Tukey’s *post hoc* test).

The following claudins were amplified in the intestine of seabream: *cldnb*, *cldnk*, *cldn3*, *cldn5*, *cldn7b*, *cldn12*, *cldn15*, *cldn24*, and *cldn34*. Figure 4 shows the complete expression analysis of this set of claudins. Regional relative mRNA expression analysis of *claudins* in control fish revealed that five claudin genes out of 9 were significantly higher in anterior than posterior intestine, for example, *cldn3*, *cldn5*, *cldn12*, and *cldn15*. In contrast, only a single *claudin* of those analyzed, for example, *cldn24*, showed higher expression in posterior intestine, as no expression was detected in the anterior intestine (Figure 4H).

AFB1 feeding induced several changes in *claudin* expression. Overall, the anterior intestine was more sensitive to dietary AFB1, and the effects included upregulation, downregulation, or no impact on relative claudin expression. Specifically, relative mRNA expression of *cldnk*, *cldn5*, *cldn12*, and *cldn24* remained unchanged in response to dietary AFB1 both in the anterior or posterior intestine (Figure 4). Relative mRNA expression of *cldn3* significantly decreased at the highest level of dietary AFB1 in the anterior but not in the posterior intestine (Figure 4C). Dietary AFB1 increased expression of *cldn7b*, *cldn15*, and *cldn34* in the anterior intestine and downregulated mRNA expression of *cldnb*, *cldn7b*, and *cldn15* in the posterior intestine but not in a concentration-dependent manner (Figures 4A,E,G,I).

**Figure 5:**
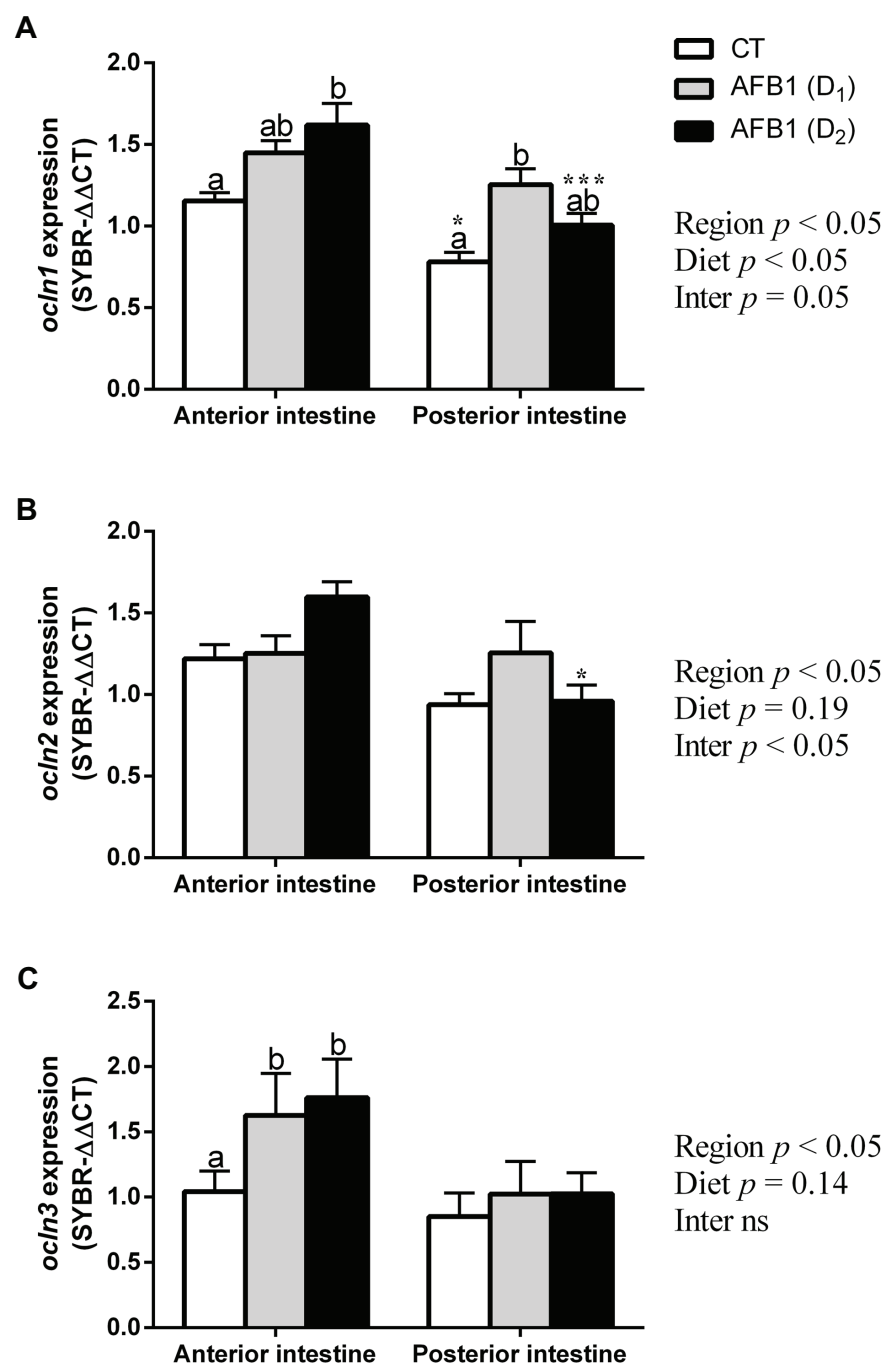
Gene expression of *ocln1* **(A)**, *ocln2* **(B)**, and *ocln3* **(C)**, in the anterior (AI) and posterior intestine (PI) of *S. aurata* juveniles fed with different experimental diets (CT, control; D_1_, 1 mg- and D_2_, 2 mg-AFB1 kg^−1^ fish feed) for 85 days. Data are presented as mean ± SEM (n = 8–10). Further details in legend Figure 4.

Occludin expression, for example, *ocln1*, *ocln2*, and *ocln3*, were observed both in the anterior and posterior intestine of the seabream (Figure 5). There is no clear anterior–posterior pattern of expression in control fish, as seen for some claudins. However, dietary AFB1 induced several changes in occludin expression. In the anterior intestine, *ocln1* expression was upregulated by AFB1 in a concentration-dependent manner, while in the posterior intestine showed an inverse U-shaped relationship with AFB1 doses (Figure 5A). However, the relative mRNA expression of *ocln2* was insensitive to dietary AFB1, although there is a trend toward increased expression at the highest dose of AFB1 in the anterior intestine (Figure 5B). In contrast, mRNA expression of *ocln3* was upregulated in anterior intestine but without significant changes in posterior intestine (Figure 5C).

##### Histopathology

**Figure 6:**
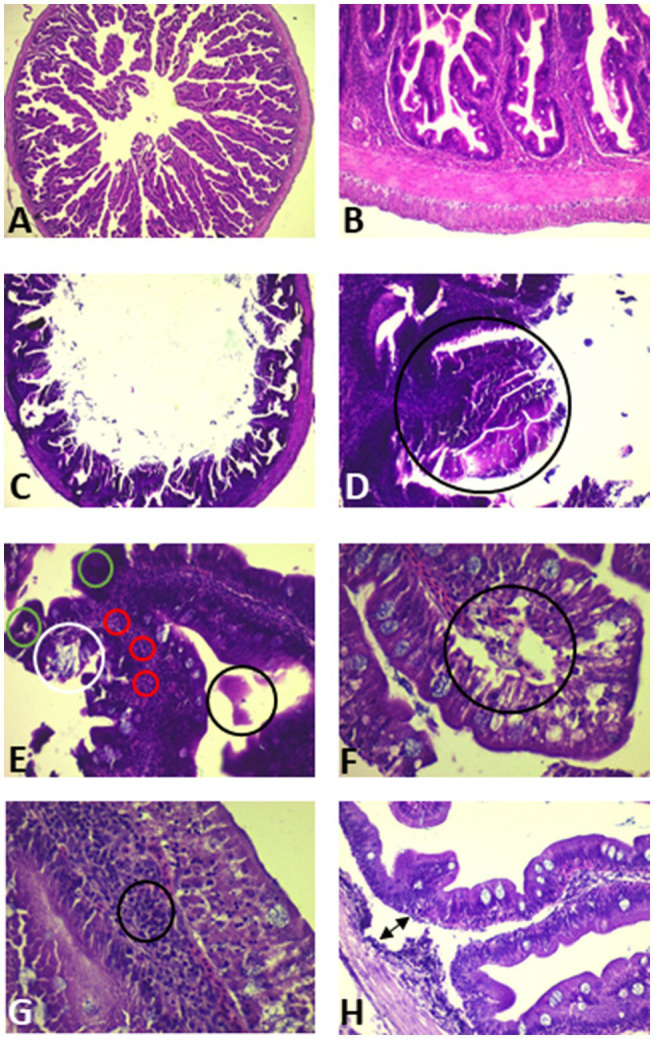
Photomicrograph of the anterior intestine (AI) of *S. aurata* juveniles fed with control diet **(A,B)** and with the highest dose of AFB1 (2 mg AFB1 kg^−1^ fish feed) for 85 days **(C–H)**. **(A)** AI of the control group (H&E 2x). **(B)** AI showing different intestinal layers and villi in detail (H&E 10x). **(C)** AI of *S. aurata* juveniles exposed to AFB1 (H&E 2x). **(D)** AI showing in detail: necrosis, shortening of villi, and hypoplasia of goblet cells (GC; black circle; H&E 10x); **(E)** necrosis of epithelial villi (green circles), *villus* sloughing (white circle), hyperemia in submucosa layer (red circles), and exudate in the intestinal lumen (black circle; H&E 20x); **(F)** oedema in the apical end of the *villus* (black circle; H&E 40x); **(G)** infiltration of mononuclear cells in mucosa submucosa layer of a *villus* (H&E 40x); and **(H)** subepithelial spaces (arrow; H&E 20x).

The microscopical examinations showed no apparent lesions in the control group (Figures 6A,B). However, the intestine of seabream with dietary exposure to AFB1 presented villi shortening, a diminishing or hypoplasia of goblet cells, and necrosis in the epithelial lining of the intestinal villi (Figures 6C,D). The microscopic examination of intestinal tissue also revealed villi sloughing with the presence of exudate in the intestinal lumen (Figure 6E), edema (Figure 6F), and infiltration of mononuclear cells in mucosa and submucosa layers (Figure 6G). Finally, separation of mucosa and submucosa layers with the development of subepithelial spaces was also observed (Figure 6H).

## Discussion

The present study shows that AFB1 has an impact on intestinal physiology in the seabream. An observation in line with our previous work in this species showing adverse effects of AFB1 on seabream growth, disruption of metabolism, and tissue integrity, for example, liver (Barany et al., 2020). Here, our experimental approach was twofold; on the one hand, we used tissues from healthy fish to assess the immediate effects of AFB1 *in vitro* in Ussing chambers. On the other hand, we assessed the intestinal response to dietary AFB1 using fish obtained from the same experimental procedure employed by Barany et al. (2020).

An *in vitro* experiment using Ussing chambers was devised to test the putative direct effects of AFB1 on the anterior intestine of fish without previous contact with the toxin. Although the advantage of this approach is that intestinal explants maintain the original function and architecture of the tissue, they have a limited life span of ~4 h (Fuentes et al., 2006). The concentrations used *in vitro* were in the low micromolar range (e.g., 8 and 16 μM of apical AFB1). These values were derived from calculations of the dietary leaching of AFB1 used in feeding experiments (Barany et al., 2020), considering feed AFB1 content (1 or 2 mg/kg of feed), feeding rates, intestinal fluid volume, and putative residence time of food (Gilannejad et al., 2019). Short-term exposure to apical AFB1 at these levels did not affect the primary markers of tissue integrity TER or intestinal permeability. Our results contrast with previous studies in human colon carcinoma Caco-2 cell cultures (Romero et al., 2016), where AFB1 exposure *in vitro* significantly reduced TER but at more prolonged periods. Therefore, this apparent disparity in effect might be related to the timing of exposure or apical AFB1 doses employed since the highest concentrations in our tests were about 6-fold lower (16 vs. 100 μM) than those used in Romero et al. (2016).

Furthermore, we measured apical to basolateral fluxes to determine putative changes in the paracellular pathway. The results with FITC (4 kD) showed no significant effects in permeability. This apparent lack of direct action on paracellular permeability might be related to unchanged permeability for larger organic solutes and/or the intrinsic biology of different intestinal regions. It has been previously shown how the permeability might differ from smaller to larger organic solutes and within other intestinal regions in mice (Tanaka et al., 2015). Although we did not find statistical differences in permeability, it appears that high concentrations of apical AFB1 (16 μM) may lower the paracellular fluxes for larger molecules according to the TER increase observed in the *in vivo* feeding experiments. Similarly, an inverse relationship between gut permeability and TER has been reported in parasitized fish intestines (Sitjà-Bobadilla et al., 2019) or intestinal mycotoxicosis (Gao et al., 2018, 2020).

In contrast, long-term feeding experiments showed that dietary AFB1 modified intestinal selectivity severely by increasing TER but was without effect on I*sc*. TER is the gold standard to assess barrier integrity and function (Wijtten et al., 2011). Interestingly, *in vivo* dietary AFB1 significantly enhanced TER in the anterior but not in the posterior intestine, showing up anterior-to-posterior differences in barrier function previously reported in seabream (Carvalho et al., 2012; Gregorio et al., 2012). To our knowledge, no other fish studies have been performed in this context. Although, the increase in tissue resistance in response to dietary AFB1 is somehow surprising considering that in Caco-2 cells, AFB1 in the culture medium decreased TER after 48 h of exposure (Gao et al., 2018). However, Galarza-Seeber et al. (2016), using gut leakage of FITC in an *in vivo* chicken model, showed that the integrity of the gut barrier function was unaffected by dietary AFB1. We suggest that these apparent disparities across studies in TER may be linked to intestinal inflammation due to a rebound effect caused by a secondary response in the long-term exposure to AFB1. Therefore, eliciting a putative biphasic inflammatory response characterized by an initial transient TER decrease that precedes a subsequently TER increase if the aflatoxicosis persists. Intestinal inflammation decreases the tissue capacity for absorption and might even revert to secretion, thus causing diarrheic disorders (Schulzke et al., 2009; Verkman et al., 2013) that ultimately impair growth performance (Barany et al., 2020). Rebound effects are recurrent physiological responses in order to adjust the organism to face a new situation or as a detrimental side effect (Fenwick et al., 1999; Montserrat et al., 2007).

The GIT is the first contact point with feed components. Therefore, dietary aflatoxins are promptly absorbed into the bloodstream from the GIT (Battacone et al., 2012; Rodrigues et al., 2019). In tetrapods, the small intestine (homologous to the anterior fish intestine) has a greater surface area due to the presence of villi (Kiela and Ghishan, 2016), while in fish, it has also been reported a greater number of villi and globet cells within this same region (Verdile et al., 2020). Note that the villi are conformed mostly by absorptive cells (Kiela and Ghishan, 2016). Thus, in our feeding experiments, we assumed the anterior intestine was exposed to much higher doses of AFB1 released from the feed than the posterior since it is in this region where the AFB1 is firstly absorbed (Rodrigues et al., 2019).

In the intestine, TJs separate the paracellular space from the intestinal lumen acting as a divider of apical and basolateral domains of plasma membranes (van Itallie and Anderson, 2013), thus regulating the paracellular movement of ions, water, and small molecules. Claudins and occludins are essential elements of a functional tight junction and determine the TER of the intestinal epithelium. Therefore, the modification in barrier properties reflected by TER is tightly linked with changes in these key structural proteins of the TJ (Curry et al., 2020). To understand the underlying molecular network of altered tissue resistance in response to dietary AFB1 observed in feeding experiments in the seabream, we performed a qPCR array that included several claudins and occludins. Bearing in mind that in most studies performed to date, claudin gene mRNA expression usually reflects protein levels and can be considered a proxy for their function (Alexandre et al., 2005; Tanaka et al., 2015). In our study, dietary AFB1 had a wide range of effects on claudin and occludin effects in expression (measured by qPCR) in the seabream intestine. Specifically, it seems that the anterior intestine is predictably more responsive than the posterior intestine to AFB1 exposure, in line with the observed histopathological effects (e.g., inflammation). This observation is unsurprising since AFB1 has a low molecular weight and is assumed to be rapidly absorbed first by the proximal intestine (Rodrigues et al., 2019).

Previous studies in grass carp showed that mycotoxins downregulated barrier-forming TJ proteins expression, such as *zonula occludens 1*, *zonula occludens 2b*, *ocln*, *cldnc, cldnf, cldn7a, cldn7b*, and *cldn11*, whereas *cldn12* and *cldn15a* were upregulated (Huang et al., 2018). Based on several overexpression studies, certain *claudins*, including *cldn1*, *cldn3*, *cldn4*, and *cldn15*, increased TER, whereas *cldn2* had opposite effects (Singh and Harris, 2004; Tamura et al., 2008; Milatz et al., 2010). Additionally, certain claudins’ functions have also been linked to intestinal crypt stem cell survival, self-renewal, and epithelial differentiation (Tamura et al., 2008; Xing et al., 2020). Remarkably, differential overexpression among *claudins* possibly modified ionic paracellular selectivity, for example, Cl^−^ and Na^+^ (Alexandre et al., 2005), and organic solute fluxes (Tanaka et al., 2015).

In our study, dietary AFB1 in the seabream significantly downregulated *cldn3* in the anterior intestine and *cldnb* in the posterior intestine, while *cldn7b*, *cldn15*, and *cldn34* were upregulated in the anterior intestine. Thus, some of these changes in expression probably have physiological implications that may trigger a switch in tissue selectivity reflected by the *in vivo* TER increase and/or the apparent decrease in the intestinal tissue permeability properties for larger organic solutes (~4 kD) as previously shown by Curry et al. (2020). Similarly, previous studies have provided a relationship between TER decreased and permeability enhancement (Villarroel et al., 2009).

Few studies have investigated the role of occludin-proteins/genes in fish to the best of our knowledge. Overall, their distribution is tissue-specific, and they play a role in hydromineral balance through the regulation of epithelial tightness. Such is the case in fish, where occludins play an essential role in regulating paracellular solute movements (Chasiotis and Kelly, 2008; Tipsmark and Madsen, 2012) and show the unambiguous distribution to apical sections of osmoregulatory tissues, such as the gill, kidney, and GIT. Previous studies have correlated low protein expression with leaky epithelia, whereas high protein expression appears in tight epithelia (Chasiotis and Kelly, 2008). Occludin downregulation parallels decrease TER in Caco-2 epithelial monolayers (Musch et al., 2006; Zeissig et al., 2007).

Here we identified three different occludins, for example, *ocln1*, *ocln2*, and *ocln3* in the GIT system of seabream, without an apparent pattern of anterior–posterior expression differentiation. On analyzing the dietary effects of AFB1, we found a significant increase of relative mRNA expression of *ocln1* and *ocln3* in the anterior intestine, likely associated with the TER enhancement in response to dietary AFB1 as previously reported in cells *in vitro* with androgens (Kaitu’u-Lino et al., 2020). Note that *occludin 1b* in the human distal intestine might also play an essential role in intestinal leakage across TJs (Muresan et al., 2000). In addition, corticosteroids (e.g., cortisol) and other hormones can also directly modulate epithelial permeability characteristics reflected by changes in TER (Chasiotis et al., 2010; Trubitt et al., 2015). We believe this is not the case in our study since we found no differences in plasma cortisol in response to dietary AFB1 (Barany et al., 2021). Thus, and besides endogenous hormones, anti-nutritional factors, such as AFB1, seem to alter the intestinal barrier function properties throughout diet alone. Similar suppressive effects on occludins relative expression have also been reported in the intestine of Atlantic salmon (Moldal et al., 2018). Specifically, and among other effects, deoxynivalenol was suggested to diminish the expression of occludins in the distal intestine.

Mycotoxins also alter the functions and histomorphology of the intestine (Liew and Mohd-Redzwan, 2018; Alassane-Kpembi et al., 2019). In the present study, dietary AFB1 induced desquamation with exudate in the intestinal lumen of the anterior region, while goblet cells (GC) decreased in the epithelial lining of the intestinal villi. Previous studies on AFB1 dietary effects on fishes also observed sloughing of surface epithelial in the intestine of *Catla catla* (Andleeb et al., 2015) and described hypoplasia of GC villi sloughing in carps exposed to AFB1 (Khan et al., 2019). In our study, dietary AFB1 caused in the seabream edema in the villi, necrosis of epithelial cells, and mononuclear cells infiltration in the submucosal layer of the villi. Also, subepithelial spaces were observed due to the separation of mucosa and submucosa layers (Akinrinmade et al., 2016). Thus, we suggest some of these gut-architectural affections might be caused by the specific toxic binding of AFB1 to constituents of TJs directly exposed to the toxicant (Sonoda et al., 1999), plus that observed claudins dysregulation may also interfere with TJs signaling to regulate local cell turnover (Tanaka et al., 2015; Xing et al., 2020).

In summary, our results showed that dietary AFB1 modifies fundamental mechanisms of intestinal physiology in the seabream. AFB1 impacts intestinal histomorphology and alters barrier function. Interestingly, no short-term effects were observed, indicating that chronic exposure, and molecular alterations, are needed to reveal its impact. Indeed, we reported that long-term dietary AFB1 modifies barrier function as shown by TER measurements and scrambles the expression of key components of the tight junction, *claudins*, and *occludins*. Based on these results, we conclude that dietary AFB1 in the gastrointestinal system is the base of the previously reported growth impairment of AFB1 in the seabream (Barany et al., 2020) that later led to altered physiological stress responses to crowding densities (Barany et al., 2021). Further studies regarding the specific physiological involvement of the described *claudins* and *occludins* in the current study are warranted to fully understand the likely complex combinatorial interactions linked to intestinal permeability regulation.

## Data Availability Statement

All data generated or analyzed during this study are available as **Supplementary Material** for this article.

## Ethics Statement

The animal study was reviewed and approved by Junta de Andalucía (reference number 28-04-15-241) and by the Ethics and Animal Welfare Committee from the Spanish Government (RD53/2013). In addition, all animal manipulations were carried out in compliance with the European (Directive 2010/63/EU) and Portuguese legislation for the use of laboratory animals. All animal protocols were performed under Group-C licenses from the Direção-Geral de Alimentação e Veterinária, Ministério da Agricultura, do Mar, Ambiente e Ordenamento do Território, Portugal.

## Author Contributions

AB, JM, and JF contributed to the conception and design of the research. AB performed osmolality, enzyme activity, electrophysiology, molecular analysis, data curation, statistical analysis, and drafted manuscript. AB and SFG performed Na^+^ and permeability analysis. MO performed the histopathological examination. GM-R supervised molecular analyses and performed phylogenies. AB, MO, SFG, GM-R, JM, and JF edited and revised the manuscript. All authors contributed to the article and approved the submitted version.

## Funding

This work was funded by the Spanish Ministry of Economy and Business-MINECO (AGL2016-76069-C2-1-R) awarded to JM. The authors (AB and JM) belong to the Fish Welfare and Stress Network (AGL2016-81808-REDT), supported by the Agencia Estatal de Investigación (MINECO, Spanish Government). AB was supported by the University of Cadiz Ph.D. scholarship (PIF UCA/REC02VIT/2014). CCMar is supported by national funds from the Portuguese Foundation for Science and Technology (FCT) through project UIDB/04326/2020.

## Conflict of Interest

The authors declare that the research was conducted in the absence of any commercial or financial relationships that could be construed as a potential conflict of interest.

## Publisher’s Note

All claims expressed in this article are solely those of the authors and do not necessarily represent those of their affiliated organizations, or those of the publisher, the editors and the reviewers. Any product that may be evaluated in this article, or claim that may be made by its manufacturer, is not guaranteed or endorsed by the publisher.
