# Supplementary material for: Dysregulation of Intestinal Physiology by Aflatoxicosis in the Gilthead Seabream (Sparus aurata)
Source: Front Physiol. 2021 Dec 20;12:741192. doi: 10.3389/fphys.2021.741192 (PMC8722709; doi:10.3389/fphys.2021.741192)
Supplement: Supplementary file 1 [file Data_Sheet_1.ZIP › Supplementary_Figures (Frontiers in Physiology).docx]

Supplementary Material

Total RNA from the intestine (RIN>9) were sent to StabVida (Portugal), where an RNAseq experiment was performed by NGS mass sequencing, using the Illumina HiSeq® 2500 platform. To obtain the sequences, a new alignment was performed with the Trinity software, annotating the transcripts with the Blast2GO software (Conesa et al., 2005). Once the genes of interest were found, additional blast analyses for nucleotides and putative proteins were performed. Other genes of interest were pulled out from GenBank. Two phylogenetic analyses using NGPhylogeny.fr with default parameters (PhyML), visualizing the tree with interactive Tree of Life (iTOL; https://itol.embl.de; Supplementary material: Figures S1 and S2), were performed. These two trees were made in order to clarify the phylogenetic positions of the genes, given blast analyses with the putative proteins (from MW731470.1 and MW731472.1, on one hand, and MW731476.1, on the other hand) gave hits with Claudin-4-like and Occludin-like, respectively.

Conesa, J.A., Gotz, S., Garcia-Gomez, J.M., Terol, J., Talon, M., Robles, M., 2005. Blast2GO: a universal tool for annotation, visualization and analysis in functional genomics research, Bioinformatics 21(18), 3674–3676.





**Figure S1.** Phylogenetic tree for several Claudin-4-like protein sequences from human, mouse, frog, and few teleosts, in order to name them according to their relative positions. (*) Position for protein sequence from GenBank acc. no. MW731472.1 (*claudin k*; *cldnk*), identical to Predicted NBCI RefSeq XM_030408301.1 *Sparus aurata* *claudin-4-like* (LOC115575893), mRNA. (**) Position for protein sequence from GenBank acc. no. MW731470.1 (*claudin b*; *cldnb*), identical to Predicted NBCI RefSeq XM_030437103.1 *Sparus aurata* *claudin-4-like* (LOC115593552), mRNA. In the teleost lineage, two main clades for *Danio rerio*, one for Claudin k and another for Claudin b, can be observed.





**Figure S2.** Phylogenetic tree for several Occludin protein sequences from a few teleosts, in order to name them according to their relative positions. (*) Position for protein sequence from GenBank acc. no. KF861990.1 (*occludin 1*; *ocln1*), identical to Predicted NCBI RefSeq XM_030434673.1 *Sparus aurata* *occludin-like* (LOC115592139), mRNA. (**) Position for protein sequence from GenBank acc. no. MW731476.1 (*occludin 2*; *ocln2*), identical to Predicted NBCI RefSeq [XM_030433803.1](https://www.ncbi.nlm.nih.gov/sites/entrez?cmd=Search&db=nucleotide&term=XM_030433803.1&dopt=GenBank) *Sparus aurata* *occludin-like* (LOC115591641), mRNA. (***) Position for protein sequence from GenBank acc. no. JQ692876.1 (*occludin 3*; *ocln3*), identical to Predicted NBCI RefSeq [XM_030403521.1](https://www.ncbi.nlm.nih.gov/sites/entrez?cmd=Search&db=nucleotide&term=XM_030403521.1&dopt=GenBank) *Sparus aurata* *occludin-like* (LOC115572975), mRNA. Three main clades can be observed, from which the first one, where XP_030290533.1 from *Sparus aurata* is located, is the only one with five out of five PR01258 Motifs from Prints database for Occludin when searching in InterPro (Blum et al., 2020).

Blum, M., Chang, H., Chuguransky, S., Grego, T., Kandasaamy, S., Mitchell, A., Nuka, G., Paysan-Lafosse, T., Qureshi, M., Raj, S., Richardson, L., Salazar, G.A., Williams, L., Bork, P., Bridge, A., Gough, J., Haft, D.H., Letunic, I., Marchler-Bauer, A., Mi, H., Natale, D.A., Necci, M., Orengo, C.A., Pandurangan, A.P., Rivoire, C., Sigrist, C.J.A., Sillitoe, I., Thanki, N., Thomas, P.D., Tosatto, S.C.E., Wu, C.H., Bateman, A., Finn, R.D., 2020. The InterPro protein families and domains database: 20 years on. Nucleic Acids Research. doi: <https://doi.org/10.1093/nar/gkaa977>
